# Hip fracture in patients with non-dialysis chronic kidney disease stage 5

**DOI:** 10.1038/s41598-021-00157-1

**Published:** 2021-10-18

**Authors:** Chao-Hsiun Tang, Che-Yi Chou

**Affiliations:** 1 School of Health Care Administration, College of Management, Taipei Medical University, Taipei, Taiwan, ROC; 2 Division of Nephrology, Asia University Hospital, No. 222, Fuxin Rd, Wufeng Dist., Taichung, 413 Taiwan, ROC; 3 Department of Post-Baccalaureate Veterinary Medicine, Asia University, Wufeng, Taichung, Taiwan, ROC; 4 Divsion of Nephrology, China Medical University Hospital, Taichung, Taiwan, ROC

**Keywords:** Diseases, Health care, Nephrology

## Abstract

Hip fracture is a significant health problem and is associated with increased mortality. Patients with chronic kidney disease (CKD) are more at risk of hip fracture than the general population, but the hip fracture risk is not evident among non-dialysis CKD stage 5 patients. This study aims to assess the risk of hip fracture in patients with non-dialysis CKD stage 5 comparing to those with CKD stages 1–4. Patients with non-dialysis CKD stage 5 and CKD stages 1–4 were retrieved from Taiwan longitudinal health insurance database 2011–2014. All patients were followed to the end of 2018 for the development of hip fractures. We analyze the risk of hip fracture of propensity score-matched patients with CKD stage 5 compared to patients with CKD stages 1–4 using stepwise Cox regression and competing risks regression. We analyzed 5649 propensity score-matched non-dialysis CKD 1–4 patients and non-dialysis CKD 5 patients between 2011 and 2014. All patients were followed to the end of 2018, 229 (4.1%) of CKD 1–4 patients in 21,899 patient-year, and 290 (5.1%) of CKD 5 patients had hip fractures in 18,137 patient-year. CKD 5 patients had a higher risk of hip fracture than patients with CKD stages 1–4. The adjusted HR was 1.53 (95% CI 1.08–1.54) in the Cox regression with adjustments for age, gender, comorbidity, and history of fracture. In the competing risks regression, the subdistribution hazard ratio was 1.29 (95% CI 1.08–1.54). Female gender, age, history of fractures, and Charlson–Deyo comorbidity index were independently associated with increased hip fracture risks. Non-dialysis CKD 5 patients had a higher risk of hip fracture than patients with CKD stages 1–4. This association is independent of patients’ age, female gender, history of fractures, and comorbidities.

## Introduction

Hip fractures are a significant public health issue worldwide and are linked to increased morbidity, mortality, and economic burden^1^. Hip fracture has a substantial impact on patient's abilities, function, and quality of life^2^. Hip fracture incidence was higher in patients with chronic kidney disease (CKD) stages 3–5 than individuals with normal renal function^3^. CKD is associated with poor outcomes following hemiarthroplasty for hip fractures. CKD patients have higher risks of surgical complications, readmission, and mortality^4^. However, the risk of hip fracture was unclear in non-dialysis CKD stage 5 patients because only a small number of individuals and events for CKD stage 5 resulted in insufficient statistical power in the previous study^3^. This study aims to assess hip fracture risk in patients with non-dialysis CKD stage 5 (CKD 5 ND).

## Methods

The study complied with the Declaration of Helsinki. It was approved by the internal review board of Taipei Medical University (TMU-JIRB No.: N201903124). The requirement for informed consent was waived by the internal review board of Taipei Medical University since the identification information has been encrypted to ensure privacy.

### Data source

There were three databases used in this study. First, 2011–2018 Taiwan National Health Insurance Research database; Second, 2011–2015 Cancer Registry; and Third, 2011–2018 death registry.

### Study subjects

**Figure 1:**
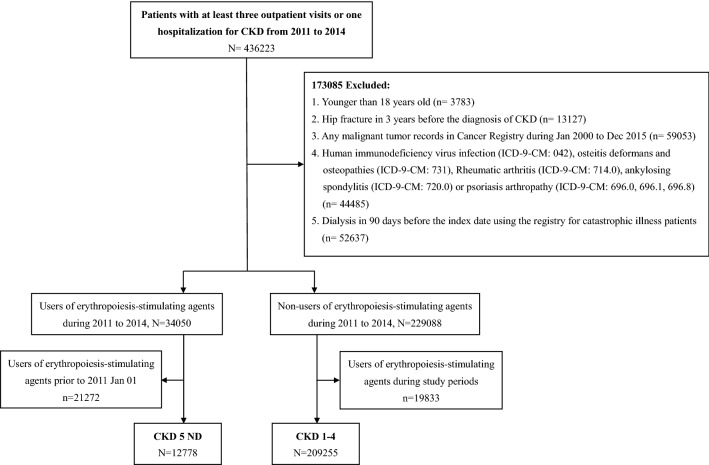
Flow chart of study design.

Inclusion criteria were patients with at least three outpatient visits or one hospitalization for CKD from 2011 to 2014 (Fig. 1). The ICD-9-CM code for CKD includes 581.9, 582.9, 584.9, 585, and 586. The date of the CKD stage 1–4 diagnosis was defined as the index date. CKD 5 ND patients were identified using the prescription of erythropoiesis-stimulating agents (ATC code B03XA01 and B03XA02) because these medications were only prescribed to patients with an estimated glomerular filtration rate less than 15 ml/min/1.73 m^2^. The date of the first erythropoiesis-stimulating agent prescription was defined as the index date. Patients who met the following criteria were excluded: (1) younger than 18 years old; (2) had any hip fracture during 3 years before the diagnosis of CKD 3; (3) any malignant tumor records in Cancer Registry during Jan 2000 to Dec 2015 (ICD-9-CM code 140-208); (4) human immunodeficiency virus infection (ICD-9-CM code 042) or osteitis deformans and osteopathy (ICD-9-CM code 731); (5) patients who had rheumatic arthritis (ICD-9-CM code 714.0), ankylosing spondylitis (ICD-9-CM code 720.0), or psoriasis arthropathy (ICD-9-CM code 696.0, 696.1, 696.8); (6) patients who received dialysis in 90 days before the index date in the registry for catastrophic illness patients. A history of fracture was defined as any vertebral fracture (ICD-9-CM code 805.2, 805.4, 805.6, 805.8, 806.2, and 806.4), upper humerus fracture (ICD-9-CM code 812.0), wrist fracture (ICD-9-CM code 813.4) in 3 years before the index date.

### Outcome measurement

All patients were followed to Dec 31, 2018, to develop hip fracture, defined by any outpatient or inpatient claims records with ICD-9-CM codes of 820.0, 820.2, and 820.

### Variable definitions

We extracted the medication used for more than 14 days within one month before the index date. Medications of interest included vitamin D (ATC code A11CC), calcium acetate (ATC code V03AE07), calcium carbonate (ATC code A12AA04, A02AC01), aluminum hydroxide (ATC code A02AB01), magnesium oxide (ATC code A02AA02), denosumab ATC code M05BX04), folic acid (ATC code B03BB01, B03BB51), vitamin B (ATC code A11E), anti-hypertension medications and anti-diabetic medications. Patients’ comorbidity was calculated using the Charlson–Deyo comorbidity index (CCI)^5^.

### Statistical analysis

Patient characteristics were expressed as number (percentage) or mean (standard deviation) where appropriate. Chi-square tests and *t* test was used to test the differences of variables between the two groups. The sensitivity analysis was performed using the propensity score matching with sex, age, history of fractures, medications, and CCI with a ratio of 1:1. The risk of hip fracture was analyzed Cox regression, and the variables with a p < 0.05 were further analyzed using stepwise Cox regression with adjustments for age, gender, comorbidity, history of fractures, and CCI. Hazard ratios (HRs) and 95% confidence interval (CIs) of HRs were calculated. The results of Cox regression were further analyzed using competing risk regression^6^. Death and dialysis were considered as competing events. Subgroup analysis was performed for age, comorbidities, and CCI. All statistical analyses were carried out using SAS statistical package, version 9.3 (SAS, Inc., NC, USA). All comparison tests were two-sided, and a *p* value of less than 0.05 was considered statistically significant.

### Ethics approval and consent to participate

This study was approved by the internal review board of Taipei Medical University (TMU-JIRB No.: N201903124).

## Results

**Figure 2:**
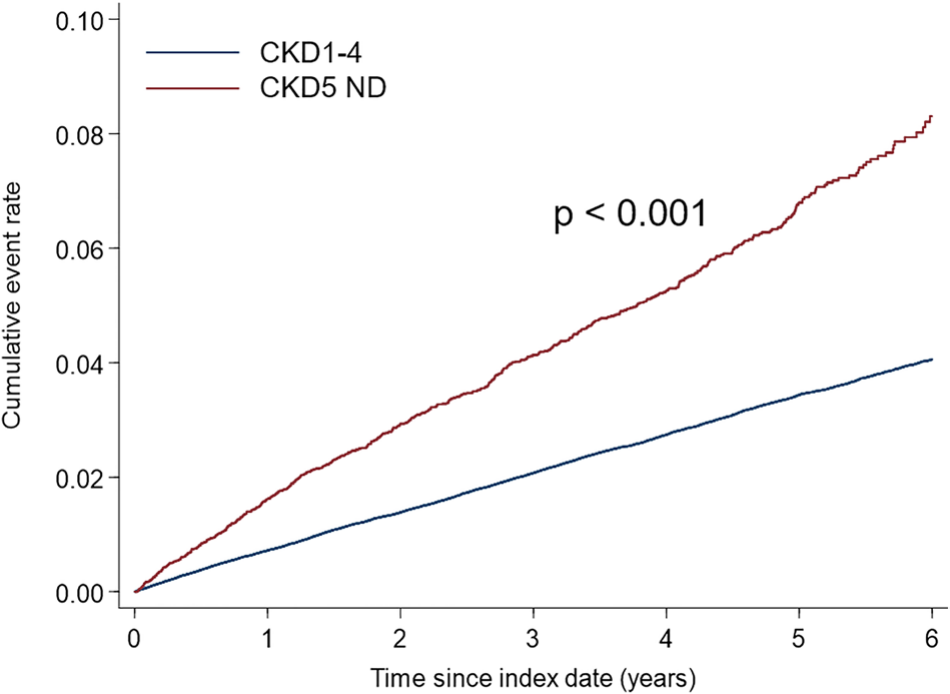
Kaplan–Meier plot of hip fracture risk in patients with chronic kidney disease stage 1–4 and chronic kidney disease stage 5 non-dialysis.

A total of 222033 patients, including 209255 non-dialysis CKD 1–4 patients and 12778 non-dialysis CKD 5 patients, were included (Table 1). The patients with CKD 5 were younger than those with CKD 1–4 (mean age 65.3 and 66.9 years, p < 0.001). The percentage of diabetes and hypertension was significantly higher in CKD 5 patients. 7664 (60%) CKD 5 patients had diabetes, and 78852 (38%) CKD 1–4 patients had diabetes. 10604 (83%) CKD 5 patients had hypertension, and 134828 (64%) CKD1-4 patients had hypertension. The CCI is higher in CKD 5 patients than CKD 1–4 patients (4.0 ± 1.8 vs. 1.7 ± 1.7, p < 0.001). 9482 (74%) CKD patients had at least three comorbidities. 2059 (16%) CKD 5 patients had vitamin D, 4362 (34%) calcium acetate, 455 (4%) ca carbonate, 934 (7%) magnesium oxide, 6944 (54%) folic acid, and 3383 (26%) vitamin B. 6179 (3.0%) of CKD 1–4 patients had hip fractures in 890302 patient-year, and 632 (4.9%) of CKD 5 patients had hip fractures in 45056 patient-year. CKD 5 patients had a higher risk of hip fracture than patients with CKD stages 1–4 (Fig. 2). We analyzed 5649 CKD 5 and CKD 1–4 patients after propensity score matching with age, gender, comorbidity, history of fractures, CCI, and medications (Table 2).

**Table 1:** Clinical characteristics of all patients.

Variables	All patients		CKD 1–4		CKD 5	
	N = 222,033		N = 209,255		N = 12,778	
Male	133,626	60%	126,706	61%	6920	54%
**Age**	66.8 ± 15.0		66.9 ± 15.0		65.3 ± 13.9	
18–44	18,010	8%	17,020	8%	990	8%
45–64	73,873	33%	68,943	33%	4930	39%
≧ 65	130,150	59%	123,292	59%	6858	54%
**Comorbidity**						
Diabetes	86,516	39%	78,852	38%	7664	60%
Hypertension	145,432	66%	134,828	64%	10,604	83%
History of fracture	12,164	5.5%	11,498	5.5%	666	5.2%
**CCI**	1.9 ± 1.8		1.7 ± 1.7		4.0 ± 1.8	
CCI = 0	62,371	28%	62,228	30%	143	1%
CCI = 1	46,704	21%	46,644	22%	60	0%
CCI = 2	45,467	20%	42,374	20%	3093	24%
CCI ≧ 3	67,491	30%	58,009	28%	9482	74%
**Medications**						
Anti-HTN	139,389	63%	129,150	62%	10,236	80%
Anti-DM	79,431	36%	72,337	35%	7094	83%
Vitmin D	3300	1%	1241	1%	2059	16%
Ca acetate	7842	4%	3480	2%	4362	34%
Ca carbonate	660	0%	205	0%	455	4%
AlOH3	481	0%	392	0%	89	1%
MgO	25,092	11%	24,158	12%	934	7%
Denosumab	474	0%	447	0%	27	0%
Folic acid	22,048	10%	15,104	7%	6944	54%
Vitmin B	30,681	14%	27,298	13%	3383	26%

*CCI* Charlson–Deyo comorbidity index, *HTN* hypertension, *DM* diabetes mellitus, *AlOH*_*3*_ aluminum hydroxide, *MgO* magnesium oxide.

**Table 2:** Clinical characteristics of patients after propensity score matching.

Variables	CKD 1–4		CKD 5		p
	N = 5649		N = 5649		
Male	3258	58%	3258	58%	1.00
**Age**	69.4 ± 11.9		69.4 ± 11.9		1.00
18–44	142	3%	142	3%	–
45–64	1786	32%	1786	32%	–
≧ 65	3721	66%	3721	66%	–
**Comorbidity**					
Diabetes	3545	63%	3555	63%	0.85
Hypertension	4872	86%	4876	86%	0.91
History of fractures	170	3%	170	3.0%	1.00
**CCI**	3.6 ± 1.6		4.1 ± 1.8		1.00
CCI = 0	63	1%	63	1%	–
CCI = 1	30	1%	30	1%	–
CCI = 2	1134	20%	1134	20%	–
CCI ≧ 3	4422	78%	4422	78%	–
**Medications**					
Anti-HTN	3305	59%	3305	59%	1.00
Anti-DM	4738	84%	4738	84%	1.00
Vitmin D	101	2%	164	3%	< 0.01
Ca acetate	443	8%	592	10%	< 0.01
Ca carbonate	2	0%	2	0%	1.00
AlOH_3_	445	8%	594	11%	< 0.01
MgO	2	0%	9	0%	0.04
Denosumab	473	8%	422	7%	0.08
Folic acid	5	0%	5	0%	1.00
Vitmin B	2057	36%	1844	33%	< 0.01

*CCI* Charlson–Deyo comorbidity index, *HTN* hypertension, *DM* diabetes mellitus, *AlOH*_*3*_ aluminum hydroxide, *MgO* magnesium oxide.

The adjusted HR of CKD 5 was 1.53 (95% CI 1.29–1.83, p < 0.01), while patients with CKD stages 1–4 were considered as references (Table 3). The risk of hip fracture was increased with age and number of comorbidity. The adjusted HR of patients aged at least 65 years was 3.23 (95% CI 2.54–4.09) when patients younger than 65 were considered the reference. The adjusted HR of diabetes and hypertension were 1.22 (95% CI 1.02–1.47) and 1.10 (95% CI 0.86–1.43). The adjusted HR of the history of fracture was 2.38 (95% CI 1.21–1.89). Compared to the patients with CCI 0–2, the adjusted HR of patients with a CCI ≥ 3 was 1.51 (95% CI 1.21–1.89). None of the medications were associated with a decreased risk of hip fracture.

**Table 3:** Results of stepwise Cox regression and competing risks regression after propensity score matching.

Variables	Cox regression				Competing risks regression			
	aHR	p	95% CI		sHR	p	95% CI	
CKD 5	1.53	< 0.01	1.29	1.83	1.29	0.01	1.08	1.54
Female	1.51	< 0.01	1.27	1.79	1.33	< 0.01	1.11	1.58
**Age**								
< 65	Ref	–	–	–	Ref	–	–	–
≥ 65	3.23	< 0.01	2.54	4.09	1.22	0.05	1.00	1.48
**Comorbidity**								
Diabetes	1.22	0.03	1.02	1.47	–	–	–	–
Hypertension	1.10	0.44	0.86	1.43	–	–	–	–
History of fractures	2.38	< 0.01	1.64	3.46	1.57	0.02	1.08	2.31
**CCI**								
CCI 0–2	Ref	–	–	–	Ref	–	–	–
CCI ≥ 3	1.51	< 0.01	1.21	1.89	1.31	0.02	1.04	1.64
**Medications**								
Anti-HTN	1.11	0.41	0.87	1.40	–	–	–	–
Anti-DM	1.12	0.20	0.94	1.34	–	–	–	–
Vitamin D	1.11	0.70	0.65	1.89	–	–	–	–
Ca	1.05	0.76	0.78	1.40	–	–	–	–
MgO	1.52	< 0.01	1.16	1.99	–	–	–	–
Denosumab	2.58	0.34	0.36	18.36	–	–	–	–
Folic acid	1.31	< 0.01	1.10	1.56	–	–	–	–
Vitamin B	1.33	0.01	1.08	1.63	–	–	–	–

*aHR* adjusted hazard ratio, *sHR* subdistribution hazard ratio, *CCI* Charlson–Deyo comorbidity index, *HTN* hypertension, *DM* diabetes mellitus, *Ca* calcium carbonate or calcium acetate, *AlOH*_*3*_ aluminum hydroxide, *MgO* magnesium oxide.

The subdistribution HR of CKD 5 was 1.29 (95% 1.08–1.54) in the competing risks regression. The subdistribution HR of the history of fracture was 1.57 (95% CI 1.08–2.31), female gender 1.33 (95% CI 1.11–1.58), age ≥ 65 1.22 (95% CI 1.00–1.48, age < 65 as the reference), CCI ≥ 3 1.31 (95% CI 1.04–1.64, CCI 0–2 as the reference). Medication for diabetes, hypertension was not significant associated with the risk of hip fracture. Magnesium oxide, denosumab, folic acid, and vitamin B were associated with increased hip fracture risks.

In subgroup analysis of CKD 5 patients vs CKD 1–4 patients (Table 4), patients younger than 65 was associated with an adjusted HR (aHR) of 1.80 (95% CI 1.16–2.82) in Cox regression with adjustments for age, gender, diabetes, and Charlson–Deyo comorbidity index. The aHR of patients at least 65 was aHR 1.49 (95% CI 1.23–1.80), non-diabetic patients aHR 1.45 (95% CI 1.09–1.96), diabetic patients aHR 1.75 (95% CI 1.31–2.35), hypertensive patients aHR 1.54 (95% CI 1.26–1.89), patients with a CCI of 0–2 aHR 2.02 (95% CI 1.33–3.08), and patients with a CCI ≥ 3 aHR 1.44 (95% CI 1.19–1.74).

**Table 4:** Results of subgroup analysis in patients with chronic kidney disease stage 5 vs those with chronic kidney disease stage 1–4 in Cox regression with adjustments for age, gender, diabetes, and Charlson–Deyo comorbidity index.

Variables	aHR	p	95% CI	
**Age**				
< 65	1.80	0.01	1.16	2.82
≥ 65	1.49	< 0.01	1.23	1.80
**Diabetes**				
No	1.45	0.01	1.08	1.96
Yes	1.75	< 0.01	1.31	2.35
**Hypertension**				
No	1.05	0.89	0.55	2.00
Yes	1.54	< 0.01	1.26	1.89
**CCI**				
CCI 0–2	2.02	< 0.01	1.33	3.08
CCI ≥ 3	1.44	< 0.01	1.19	1.74

*aHR* adjusted hazard ratio, *CI* confidence interval, *CCI* Charlson–Deyo comorbidity index.

## Discussion

The increased risks of hip fracture in patients with CKD are also supported by previous studies^7,8^. We further demonstrate a high hip fracture in non-dialysis CKD 5 patients compared to patients with CKD stages 1–4 in Cox regression. The application of Cox regression in this study was supported by test for the proportional hazard assumption (ESM Appendix Table 1). The association between CKD and hip fracture may be explained by CKD-related mineral and bone disorders^9,10^ and CKD-associated frailty^11^. Parathyroid hormone, calcium, phosphate are the critical component of mineral and bone disorders. Calcium-based phosphate binders are prescribed to patients with hyperphosphatemia. Vitamin D was prescribed to hyperparathyroidism patients. Patients with hyperphosphatemia and hyperparathyroidism had a high risk of hip fracture. We analyzed medications, including calcium-based phosphate binders, vitamin D, and denosumab. However, none of these medications were significantly associated with hip fracture. Denosumab is covered by Taiwan health insurance in patients with compression fractures and a less than − 2.4 SD bone density. These patients had a very high risk of hip fracture. A limited number of patients had denosumab treatment and the time of follow-up is not long.

As homocysteine predicts hip fracture risk^12^, homocysteine-lowering medications such as folic acid and vitamin B12 might reduce hip fracture risks^13^. However, treatment with folic acid plus vitamin B12 did not decrease hip fracture risk in the previous studies^14^. When the interaction of CKD 5 and folic acid is considered, folic acid is associated with reduced hip fracture risks in this study before the propensity score matching. However, magnesium oxide, folic acid, vitamin B were associated with an increased risk of hip fracture after propensity score matching. We also identify other hip fracture risk factors, including female gender, age, and the number of comorbidities. These findings were also supported by previous studies^15–18^.

There were some limitations to the study. First, laboratory data is not available in Taiwan's longitudinal health insurance database, and we can not identify patients’ CKD stages 1–4 using an individual’s renal function. We cannot analyze the association of parathyroid hormone, calcium, phosphate, and hip fracture. Second, cinacalcet and non-calcium-containing phosphate binders on hip fractures are not examined because health insurance does not cover these medications and therefore are not recorded. Third, immortal bias may be present in the effect of folic acid on hip fractures as patients who died during follow-up were censored in the analysis. Fourth, erythropoiesis-stimulating agents may not be prescribed in CKD 5 patients with a hemoglobin of more than 11 g/dl. 12.6% of CKD 5 patients^19^ may be classified as CKD stages 1–4 patients in the analysis because we use erythropoiesis-stimulating agents to identify CKD 5 patients.

## Conclusion

Nondialysis CKD stage 5 patients had increased hip fracture risks compared to CKD stages 1–4. Female gender, patients’ age, history of fractures, diabetes, and Charlson–Deyo comorbidity index are independently associated with increased hip fracture risks.

## Supplementary Information

Supplementary Information.

## Data Availability

The datasets used and analyzed during the current study are available from the corresponding author on reasonable request.

## References

[1] Hernlund E (2013). Osteoporosis in the European Union: medical management, epidemiology and economic burden. A report prepared in collaboration with the International Osteoporosis Foundation (IOF) and the European Federation of Pharmaceutical Industry Associations (EFPIA). Arch. Osteoporos..

[2] Dyer SM (2016). A critical review of the long-term disability outcomes following hip fracture. BMC Geriatr..

[3] Robertson L (2018). Hip fracture incidence and mortality in chronic kidney disease: The GLOMMS-II record linkage cohort study. BMJ Open.

[4] Lin SJ (2020). Effects of chronic kidney disease on hemiarthroplasty outcomes for fragility hip fracture in diabetic patients: A Nationwide Population-Based Observational Study. J. Arthroplast..

[5] Azzalini L (2019). A disease-specific comorbidity index for predicting mortality in patients admitted to hospital with a cardiac condition. CMAJ.

[6] Wang W, Wang W, Mosley TH, Griswold ME (2017). A SAS macro for the joint modeling of longitudinal outcomes and multiple competing risk dropouts. Comput. Methods Programs Biomed..

[7] Naylor KL (2014). The three-year incidence of fracture in chronic kidney disease. Kidney Int..

[8] Perez-Saez MJ (2015). Increased hip fracture and mortality in chronic kidney disease individuals: The importance of competing risks. Bone.

[9] Pimentel A, Urena-Torres P, Zillikens MC, Bover J, Cohen-Solal M (2017). Fractures in patients with CKD-diagnosis, treatment, and prevention: A review by members of the European Calcified Tissue Society and the European Renal Association of Nephrology Dialysis and Transplantation. Kidney Int..

[10] Erratum: Kidney Disease: Improving Global Outcomes (KDIGO) CKD-MBD Update Work Group (2017). KDIGO 2017 Clinical practice guideline update for the diagnosis, evaluation, prevention, and treatment of chronic kidney disease-mineral and bone disorder (CKD-MBD). Kidney Int. Suppl. 2017;7:1–59. Kidney Int. Suppl..

[11] Ho JQ, Verghese J, Abramowitz MK (2020). Walking while talking in older adults with chronic kidney disease. Clin. J. Am. Soc. Nephrol..

[12] Ao M (2019). Relationship between homocysteine, folate, vitamin B12 and physical performance in the institutionalized elderly. J. Nutr. Sci. Vitaminol. (Tokyo).

[13] Fratoni V, Brandi ML (2015). B vitamins, homocysteine and bone health. Nutrients.

[14] Garcia Lopez M (2017). B vitamins and hip fracture: Secondary analyses and extended follow-up of two large randomized controlled trials. J. Bone Miner. Res..

[15] Runesson B (2020). Fractures and their sequelae in non-dialysis-dependent chronic kidney disease: The Stockholm CREAtinine Measurement project. Nephrol. Dial. Transpl..

[16] Whitlock RH (2019). The Fracture Risk Assessment Tool (FRAX(R)) predicts fracture risk in patients with chronic kidney disease. Kidney Int..

[17] Desbiens LC, Goupil R, Madore F, Mac-Way F (2020). Incidence of fractures in middle-aged individuals with early chronic kidney disease: A population-based analysis of CARTaGENE. Nephrol. Dial. Transpl..

[18] Goto NA (2020). The association between chronic kidney disease, falls, and fractures: A systematic review and meta-analysis. Osteoporosis Int.

[19] Nephrology, T. S. O. Annual report on kidney disease in Taiwan. https://www.tsn.org.tw/UI/L/TWRD/ebook_2020%E5%B9%B4%E5%A0%B1.pdf (2020).

