# Supplementary material for: Hip fracture in patients with non-dialysis chronic kidney disease stage 5
Source: Sci Rep. 2021 Oct 18;11:20591. doi: 10.1038/s41598-021-00157-1 (PMC8523559; doi:10.1038/s41598-021-00157-1)
Supplement: Supplementary file 1 — Supplementary Information. [file 41598_2021_157_MOESM1_ESM.docx]

**Appendix**

Table 1. Test of proportional-hazards assumption after propensity score matching

| Variables | rho | chi2 | p-value |
| --- | --- | --- | --- |
| CKD type | 0.02547 | 0.34 | 0.5585 |
| Sex | 0.06631 | 2.24 | 0.1347 |
| Age 65+ | 0.05719 | 1.68 | 0.1948 |
| History of fractures | -0.08084 | 3.39 | 0.0655 |
| CCI≧3 | 0.01854 | 0.18 | 0.6709 |
| MgO | -0.02635 | 0.36 | 0.5488 |
| **Global test** | **-** | 7.37 | **0.2883** |
